# Helium Saver Mode With Nitrogen Make‐Up Cooling Gas for GC–MS With Cold EI

**DOI:** 10.1002/jms.70081

**Published:** 2026-07-02

**Authors:** Aviv Amirav

**Affiliations:** ^1^ School of Chemistry Tel Aviv University Tel Aviv Israel; ^2^ Aviv Analytical Ltd Hod Hasharon Israel

**Keywords:** Cold EI, GC column carrier gas, GC–MS, Helium Saver mode, nitrogen carrier gas

## Abstract

Cold EI uses helium as the column carrier and cooling make‐up gases at ~50 mL/min. In the rare case that helium supply could be temporarily interrupted, nitrogen or hydrogen can be used, but hydrogen leads to decomposition at the GC injector, and thus, nitrogen is preferred. However, nitrogen leads to longer analysis time and/or reduced separation. We describe the use of helium as the column carrier gas and nitrogen as the cooling make‐up gas. In this “Helium Saver” mode, the helium consumption is as in standard EI and nitrogen can be used alone in stand‐by mode for further helium saving.

## 1 Introduction

GC–MS with Cold EI is based on interfacing the GC and MS with a supersonic molecular beam (SMB) along with electron ionization of vibrationally cold sample compounds in the SMB in a fly‐through ion source (hence the name Cold EI) [1, 2, 3, 4, 5, 6, 7, 8]. GC–MS with Cold EI improves all the central performance aspects of GC–MS: enhanced molecular ions, improved sample identification, significantly extended range of compounds amenable for analysis, uniform response to all analytes, faster analysis, greater selectivity, and lower limits of detection [4, 5, 6, 7]. Thus, it is the best GC–MS technology. Cold EI was initially developed in 1990 [1, 2], it was recently reviewed in [4], and a book on GC–MS with Cold EI was published [5].

However, a few claim that Cold EI suffers from high helium consumption. Thus, we explored the details of the use of Cold EI with hydrogen and nitrogen [6]. Hydrogen is “ultimate” inert with Cold EI at the ion source due to its fly‐through configuration, but it suffers possible degradation at the GC injector and thus should be avoided unless for a few specific applications. Consequently, nitrogen should be preferred as the helium alternative gas for Cold EI as demonstrated and discussed in [6]. However, the use of nitrogen as the GC carrier gas either prolongs (doubles) the analysis time and/or reduces the separation by a factor of about 1.5.

Thus, we explored the use of helium as the column carrier gas and nitrogen as the Cold EI cooling make‐up gas. This newly explored combination is named Helium Saver mode, as the helium gas consumption is exactly as in standard EI. Furthermore, nitrogen can be used alone in a stand‐by (night) mode for helium saving beyond the standard EI helium consumption.

The subject of helium shortages or its interrupted supply has led to several different attempts for helium conservation methods and instrumentation as cited in [6]. However, and as discussed in [6]:

1. **There is no intrinsic helium shortage.** Thus, due to the advantages of using helium, we should continue its use while purchasing additional spare cylinders “just in case” a temporary shortage will occur and induce a short‐term supply interruption.
2. **Helium gas is not expensive.** Currently, the price of 99.999% helium at GC–MS laboratories is around $20–40 per cubic meter. Thus, for 40 min analysis time at an average of 20 mL/min, the helium consumption is under 0.8 L, which costs 2.4 cents, and that is negligible compared to other costs of the analysis such as operator salary, system amortization, electricity, city tax, maintenance, and other consumables.
3. **Cold EI Helium consumption is about that of Standard EI.** While GC–MS with Cold EI consumes 50 mL/min helium as make‐up gas for generating a “cold supersonic molecular beam,” typical analysis times are below 10 min, and with GC oven cooling time, it is in total about 14 min. Thus, at 70 mL/min total GC–MS with Cold EI helium consumption per minute, the helium consumption per analysis is 1 L with cost of 3 cents, which is negligible. At night or “stand‐by” mode, the helium consumption is under 10 mL/min in both standard EI and Cold EI. Thus, one helium cylinder with 10 m^3^ that costs $300 serves one GC–MS system for a year (if there are no leaks) at the price of under $1/day.

However, for those few that wish to have for Cold EI an alternative mode of operation with helium consumption per minute the same as or below that of standard EI, we developed and explored the Helium Saver mode. In this Helium Saver mode, helium is used as the column and GC injector gas, the same as in standard EI, while nitrogen is used as the Cold EI cooling gas.

## 2 Experimental

We used the 5975‐SMB GC–MS with Cold EI system that is based on the combination of an Agilent 7890A GC + 5975B MSD (Agilent Technologies, Santa Clara, CA, USA) with the Aviv Analytical supersonic molecular beam interface and its dual‐cage fly‐through ion source (Aviv Analytical LTD, Hod Hasharon, Israel). The details of the Cold EI system and configuration are given in references [4, 6, 7, 8].

GC separations as described in this note were performed with a 15 m column with 0.32 mm I.D., 0.1 μm DB1‐HT film (Agilent Technologies, Folsom, CA, USA) with a 2 mL/min helium column flow rate. The GC oven temperature was ramped from 50°C to 300°C at 40°C/min and held at 300°C for 1.75 min for a total analysis time of 8 min. A standard test mixture of 10 ng/μL each hexadecane (n‐C_16_H_32_), methyl stearate, cholesterol, and dotriacontane (n‐C_32_H_66_) was analyzed. This sample was injected split at a 9:1 ratio to provide 1 ng each compound on‐column. Nitrogen make‐up gas was provided from a 10 m^3^ cylinder at 99.999% purity via copper tubing, and it was filtered by a carbon gas filter.

## 3 Results—GC–MS With Cold EI Operation With Nitrogen Make‐Up Gas

**FIGURE 1:**
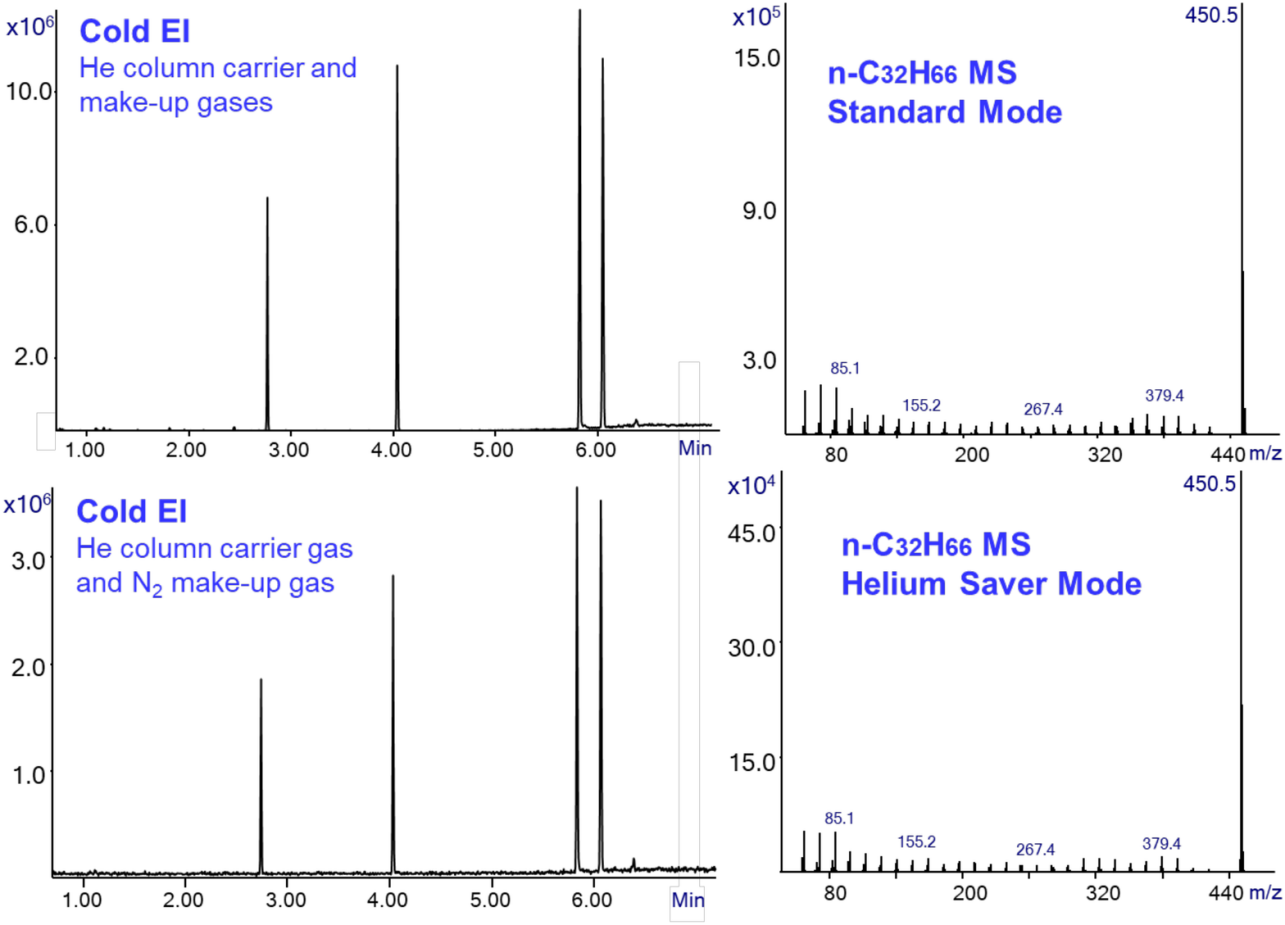
A comparison of GC–MS with Cold EI analyses of a test mixture of 1 ng on‐column each n‐C_16_H_32_, methyl stearate, cholesterol, and n‐C_32_H_66_ (10 μg/mL each with split 9:1). The upper mass chromatogram and n‐C_32_H_66_ Cold EI mass spectrum were obtained with the usual 2 mL/min helium column flow rate and 48 mL/min helium make‐up gas flow rate. The bottom mass chromatogram and n‐C_32_H_66_ Cold EI mass spectrum were obtained with the same 2 mL/min helium column flow rate and 8 mL/min nitrogen make‐up gas flow rate. Note the similarity of the mass chromatograms and Cold EI mass spectra. The retention times are 2.75, 4.04, 5.84, and 6.08 min in order of elution times for n‐C_16_H_32_, methyl stearate, cholesterol, and n‐C_32_H_66_, respectively.

We evaluated the operation of GC–MS with Cold EI with helium as the column carrier gas and nitrogen as the cooling make‐up gas (Helium Saver mode). As described in references [6, 9, 10], the vibrational cooling efficiency per nitrogen make‐up flow rate is better than with helium since it has a bigger mass that is closer to that of the sample compound [9, 10], and thus, 8 mL/min was enough. In Figure 1, we compare the analysis of our standard test mixture that includes 1 ng on‐column each hexadecane (n‐C_16_H_34_), methyl stearate, cholesterol, and dotriacontane (n‐C_32_H_66_) in order of their elution times. The upper mass chromatogram and Cold EI mass spectrum of n‐C_32_H_66_ were obtained with 15 mm, 0.32 mm ID column, with 2 mL/min helium column flow rate and 48 mL/min helium make‐up gas flow rate, while the bottom mass chromatogram and n‐C_32_H_66_ mass spectrum were obtained with the Helium Saver mode with the same column and with the same 2 mL/min helium column flow rate (same column and system) and 8 mL/min nitrogen make‐up flow rate.

Figure 1 demonstrates the concept of Helium Saver mode with helium column flow and nitrogen serving as the cooling make‐up gas. Furthermore, this figure and data demonstrate several important Cold EI benefits that are retained with the Helium Saver mode, including:

1. Good vibrational cooling that results in enhanced molecular ions is preserved with nitrogen make‐up gas as demonstrated for n‐C_32_H_66,_ yet with a relatively low nitrogen flow rate.
2. The separation in the Helium Saver mode is the same as in regular Cold EI with helium gas in both column and make‐up. This is unlike Cold EI operation with nitrogen as the column carrier gas as described in [6].
3. The signal in Helium Saver mode is reduced by about a factor of 3.5, as shown in the Y axis in Figure 1, and as in full operation with nitrogen [6]. This signal reduction is mostly due to reduced jet separation efficiency, since nitrogen is seven times heavier than helium.
4. The response uniformity feature of Cold EI is retained in the Helium Saver mode as shown in Figure 1. This is in contrast with standard EI analysis of this test mixture as shown in [4, 7].
5. No peak tailing is observed in Cold EI with the Helium Saver mode, in contrast to standard EI analysis of this test mixture, as shown in [4, 7]
6. Very high ratio of peaks to baseline is shown, in contrast to standard EI analysis of this test mixture, as shown in [4, 7].
7. Fast analysis is demonstrated in the Helium Saver mode, which is four times faster than in standard EI [4, 7]. This is the outcome of using a 15 m column with a 2 mL/min helium column flow rate.
8. The Helium Saver mode can be operated with up to about 4 mL/min helium column flow rate with the same tune method and thus serve also for some extension of the range of compounds amenable for GC–MS analysis [11].

## 4 Conclusions and Discussion

Helium remains the carrier gas of choice, even if purchasing and holding of additional spare helium gas cylinder(s) is required if one expects short‐time interruption/shortage periods. Helium provides greater sensitivity and column flow rate flexibility for the best extension of the range of compounds amenable for Cold EI analysis. However, if an unavoidable helium shortage may occur, the use of Cold EI in its demonstrated Helium Saver mode is recommended for helium saving. The transition from regular Cold EI operation into its Helium Saver mode is simple, with one valve closure (Helium to make‐up line) and one valve opening (Nitrogen to make‐up line). This mode changing can be automated with an electronic flow control (EFC). Furthermore, when a stand‐by (night) mode is selected, the system can be fully operated with nitrogen also at the injector and column, and this way helium gas is further saved even more than in systems with standard EI.

## Data Availability

The data that support the findings of this study are available from the corresponding author upon reasonable request.
